# MicroRNA and Protein Profiling of Brain Metastasis Competent Cell-Derived Exosomes

**DOI:** 10.1371/journal.pone.0073790

**Published:** 2013-09-16

**Authors:** Laura Camacho, Paola Guerrero, Dario Marchetti

**Affiliations:** 1 Departments of Pathology & Immunology, Baylor College of Medicine, Houston, Texas, United States of America; 2 Departments of Molecular & Cellular Biology, Baylor College of Medicine, Houston, Texas, United States of America; Rutgers - New Jersey Medical School, United States of America

## Abstract

Exosomes are small membrane vesicles released by most cell types including tumor cells. The intercellular exchange of proteins and genetic material via exosomes is a potentially effective approach for cell-to-cell communication and it may perform multiple functions aiding to tumor survival and metastasis. We investigated microRNA and protein profiles of brain metastatic (BM) versus non-brain metastatic (non-BM) cell-derived exosomes. We studied the cargo of exosomes isolated from brain-tropic 70W, MDA-MB-231BR, and circulating tumor cell brain metastasis-selected markers (CTC1BMSM) variants, and compared them with parental non-BM MeWo, MDA-MB-231P and CTC1P cells, respectively. By performing microRNA PCR array we identified one up-regulated (miR-210) and two down-regulated miRNAs (miR-19a and miR-29c) in BM versus non-BM exosomes. Second, we analyzed the proteomic content of cells and exosomes isolated from these six cell lines, and detected high expression of proteins implicated in cell communication, cell cycle, and in key cancer invasion and metastasis pathways. Third, we show that BM cell-derived exosomes can be internalized by non-BM cells and that they effectively transport their cargo into cells, resulting in increased cell adhesive and invasive potencies. These results provide a strong rationale for additional investigations of exosomal proteins and miRNAs towards more profound understandings of exosome roles in brain metastasis biogenesis, and for the discovery and application of non-invasive biomarkers for new therapies combating brain metastasis.

## Introduction

Exosomes are 30–100 nm membrane vesicles released by most cell types, including tumor cells, to their surrounding environment. They can be collected from body fluids, thus they have an important role as potential tumor markers and prognostic factors, providing a powerful non-invasive approach for tumor progression [1], [2], [3]. Exosomes biogenesis initiates with the formation of internal vesicles within multivesicular bodies (MVBs) by inward budding of the limiting membrane of late endosomal compartments. These MVBs then fuse with the plasma membrane, resulting in the release of exosomes into the extracellular space [4]. Although early research showed that cells use exosomes to eliminate superfluous macromolecules [5], recent advances have put forward notions of their specific biological functions, e.g., enabling cell-to-cell communication [6]. Exosomes can transfer proteins, soluble factors, RNAs, and miRNAs among cells [7], [8]. It is often noted that exosome concentrations are higher in cancer patients compared to healthy controls, and that they increase as the tumor progresses [9]. Increasing evidence suggests that tumor-derived exosomes can confer either anti-tumorigenic or pro-tumorigenic effects and these seemingly controversial effects can be the result of complex and synergistic interactions between exosomes, responding cells, and factors of the tumor microenvironment [10]. It has also been shown that part of the physiological role of exosomes is their ability to alter the microenvironment through their cargo, and that they may perform several functions aiding to tumor survival and metastasis [11]. For example, tumor-derived exosomes help to create an immunosuppressive tumor microenvironment by inducing apoptosis and impairing the function of effector T cells and NK cells [12], [13]. They also seem to contribute to the establishment of a pre-metastatic niche by enhancing angiogenesis, remodeling stromal cells, and by promoting extracellular matrix degradation [1], [14].

MicroRNAs (miRNAs) are small non-coding RNAs, found to be abnormally expressed in several types of tumors, and keenly implicated in the pathogenesis of human cancers [15]. Tumor exosome miRNA expression profiles may be indicative of disease risk, and exosome miRNAs are being investigated as possible biomarkers to predict and/or to diagnose progressive neoplastic stages [16]. Protein and miRNA profiles of melanoma versus melanocyte-derived exosomes have been studied [2], [17]. Furthermore, proteomic analysis of exosome-like vesicles derived from breast cancer cells have been developed [18]. However, to the best of our knowledge, there are no published miRNA profiles, of breast cancer cells-derived exosomes.Specifically no evidence has been presented investigating the miRNA and protein profiles of brain metastatic (BM) versus non-brain metastatic (non-BM) cancer cell-derived exosomes. The objective of this work was to characterize these profiles and compare cargo and actions of exosomes isolated from brain-colonizing variants (MDA-MB-231BR, CTC1BMSM, and 70 W) with their respective parental non-BM cell lines: MDA-MB-231P, CTC1P and MeWo.

## Materials and Methods

### Cell Lines

Human brain microvascular endothelial cells (HBMEC) were obtained following isolation from brain capillaries and cultured as previously described [19]. MDA-MB-231P (231P for brevity) and the brain metastatic variant MDA-MB-231BR (231BR for brevity) were provided by Dr. Patricia Steeg (The National Cancer Institute, Bethesda, MD) [19]. CTC1P (circulating tumor cell parental) and CTC1BMSM (circulating tumor cells possessing a brain metastasis-selected markers profile) were recently established by their isolation directly from blood of a breast cancer patient [20]. The brain metastasizing human 70 W melanoma cell line is a wheat germ agglutinin-resistant variant derived from the MeWo melanoma cell line [21]. Cells were cultured in Dulbecco’s Modified Eagle Medium plus F12 (DMEM/F12) supplemented with 10% fetal bovine serum (FBS) and 1% penicillin/streptomycin (Invitrogen, Carlsbad, CA) at 37°C, 5% CO_2_. When cells were cultured to isolate the exosomes, they were incubated with DMEM/F12 with 1% penicillin/streptomycin supplemented with 10% exosome-depleted FBS (System Biosciences, Mountain View, CA). Cells were used at early passages and their tumorigenic abilities were confirmed periodically by experimental metastasis assays in mice.

These studies were performed per protocol approved by the Institutional Animal Care and Use Committee (IACUC) of Baylor College of Medicine, and included all steps to ameliorate suffering as well as methods of animals sacrifice.

### Isolation of Exosomes and Transmission Electron Microscopy

Exosome isolation was performed using the ExoQuick-TC Exosome Precipitation Solution (System Biosciences, Mountain View, CA). After 72 hours of cell culturing, 10 ml of culture media was centrifuged at 3000×*g* for 15 min to remove cells and cell debris. The supernatant was mixed with 2 ml of ExoQuick-TC, refrigerated for 16 hours, and then centrifuged at 1500×*g* for 30 min at 4°C to obtain the exosomes pellet. Pellets were resuspended in 100 µl phosphate buffered saline (PBS), and exosomes were dropped onto a formvar carbon coated nickel grid and left to dry at room temperature (25°C) for 60 min. After washing the grids with PBS, they were fixed in 2% paraformaldehyde for 10 min, washed in ddH_2_O, and contrasted by adding 2% uranyl acetate for 15 min. Samples were dried overnight (16 hr), and visualized using a transmission electron microscope (H-7500 model, Hitachi, Tokyo, Japan).

### Western Blotting Analysis

Exosome and cell pellets were dissolved in the radio-immunoprecipitation assay (RIPA) protein lysis buffer (Sigma-Aldrich, St. Louis, MO) with a cocktail of protease and phosphatase inhibitors (Roche Diagnostics, Indianapolis, IN) followed by vortex-mixing. The protein concentration was determined using the bicinchoninic acid assay (BCA) (Pierce, Waltham, MA). After boiling the samples for 5 min at 95°C with Laemmli buffer containing β-mercaptoethanol (Boston Bioproducts, Ashland, MA), 60 µg of protein were resolved by SDS-PAGE, transferred to nitrocellulose membranes (Bio-Rad, Hercules, CA), and blocked with 5% (w/v) nonfat dry milk in tris buffered saline (TBS) with 0.5% (v/v) Tween-20 before being probed with the appropriate antibodies. Proteins used as positive and negative exosome markers were CD9 (BD Biosciences, San Jose, CA, clone M-L13, 1∶150 dilution), CD63 (BD Biosciences, San Jose, CA, clone H5C6, 1∶150), CD81 (Millipore, Billerica, MA, clone I.3.3.22, 1∶500), calnexin (Abcam, Cambridge, England, ab10286, 1∶3000) and GM130 (BD Biosciences, San Jose, CA, clone 35, 1∶500). Blots were washed with TBS containing 0.5% (v/v) Tween-20 (pH 7.4), before probing with horseradish peroxidase–conjugated secondary antibodies (Santa Cruz Biotechnology 1∶4000 dilution, sc-2030 and sc-2031). Blots were then exposed to film using SuperSignal West Femto Maximum Sensitivity Substrate (Thermo Scientific, Waltham, MA).

### MiRNA PCR Array

Total RNA from exosomes was isolated using mirVana miRNA isolation kit (Life Technologies, Grand Island, NY), and 10 ng of total RNA were reverse transcribed with miScript II RT kit (Qiagen, Germantown, MD) according to manufacturers’ guidelines. 20 µl of cDNA were diluted with RNase-free water prior to the PCR reaction. Real-time PCR for mature miRNA expression profiling was developed using the SYBR Green-based human breast cancer pathway-focused miScript miRNA PCR array (Qiagen, Germantown, MD) on a StepOnePlus 96-well RT PCR instrument (Applied Biosystems, Grand Island, NY). The relative quantity of the target miRNA was normalized using miRNeasy Spike-In Control (Qiagen, Germantown, MD) that was exogenously added during RNA purification. Three independent experiments were performed. The data obtained by the StepOne Software were analyzed using the ΔΔC_T_ method of relative quantification for miScript miRNA PCR arrays with the SABiosciences PCR data analysis software. Fold change of miRNA expression from BM compared with non-BM cell lines-derived exosomes was represented in a heatmap. Fold change with a *p* value <0.05 was considered statistically significant and miRNAs with statistically significant fold changes were represented separately. This dataset was submitted to the NCBI’s Gene Expression Omnibus (GEO) repository (http://www.ncbi.nlm.nih.gov/geo.html) and is accessible through GEO Series accession number GSE48934.

### Proteomic Analysis

Cell and exosome pellets were resuspended in lysis buffer containing 1% Triton X-100, 50 mM Hepes pH 7.4, 150 mM NaCl, 1.5 mM MgCl2, 1 mM EGTA, 100 mM NaF, 10 mM Na Pyruvate, 1 mM Na3VO4, 10% glycerol, and a cocktail of protease and phosphatase inhibitors (Roche Diagnostics, Indianapolis, IN). Cell samples were centrifuged at 14,000 rpm for 20 min at 4°C to isolate the protein supernatant. Protein concentration was determined using the BCA Assay (Pierce, Waltham, MA). Samples were then normalized to a concentration of 1.5 µg/µl and 40 µl of cells and exosomes protein lysates were boiled for 5 min at 95°C with Laemmli buffer with β-mercaptoethanol (Boston Bioproducts, Ashland, MA). The samples were then submitted for Reverse Phase Protein Array to the RPPA Core Facility at MD Anderson Cancer Center (Houston, TX; see Protocol S1). Linear value from the RPPA results was used for bar graphs and calculation of protein content averages in exosomes and cells. Fold change of protein content average in cells versus exosomes was calculated and representedby a histogram. Proteins abundantly detected in exosomes (0 to 3-fold change) were classified according to their gene ontology by using the Protein Analysis Through Evolutionary Relationships Classification System (http://www.pantherdb.org). The differential protein profile of brain metastatic versus non-brain metastatic cell-derived exosomes was represented in a heatmap.

### Exosomes Labeling and Imaging

Cells were seeded on cover slips in 6-well plates and transduced overnight (16 hr) with CellLight® Tubulin-RFP (red fluorescent protein) (Life Technologies, Grand Island, NY). Exosomes were labeled using the green lipophilic fluorescent dye PKH67 (Sigma-Aldrich, St. Louis, MO) for 5 min and the reaction was stopped by the addition of exosome-depleted FBS. Cells were then incubated with labeled exosomes for 5 hr, washed with PBS, fixed with 4% paraformaldehyde for 20 min, and mounted with ProLong® Gold Antifade Reagent with DAPI (4′,6-diamidino-2-phenylindole) nuclear stain (Life Technologies, Grand Island, NY). Pictures were taken using a Nikon TE-2000 inverted fluorescence microscope.

### MTT Proliferation Assay

Non-BM cell lines were incubated on a 96-well plate (8×10^3^ cells/well) for 16 hr. Medium with or without exosomes was added to the wells to evaluate the differential proliferative potential. MTT [3-(4,5-Dimethylthiazol-2-yl)-2,5-diphenyltetrazolium bromide] was added after 3, 24 and 48 hr respectively, and incubated for 4 hr at 37°C. Dimethyl sulfoxide (DMSO) was used as a solubilizing agent and absorbance of the wells was read at 540 nm. All experiments were performed in triplicates and the mean absorbance and standard deviations (SDs) were calculated.

### Adhesion Assay

Human brain microvascular endothelial cells (HBMEC) [19] were seeded on a 12-wells plate (2×10^5^ cells/well) and non-BM cell lines (1×10^6^ cells) were transduced with CellLight® Tubulin-RFP (Life Technologies). Following 16 hr incubation, cells (2.5×10^5^) of non-BM cell lines with or without exosomes from their corresponding BM derivatives were plated over the HBMEC monolayer to evaluate the differential adhesive potential. Afterwards (3 hr), wells were washed with PBS to remove non-adherent cells, fixed with 4% paraformaldehyde for 20 min, and mounted with ProLong® Gold Antifade Reagent with DAPI (Life Technologies). Experiments were performed in duplicates and six fields per well were taken on a Nikon TE-2000 inverted fluorescence microscope. Adhesive cells were counted and mean adhesive cell numbers and SDs were calculated.

### Invasion Assay

Invasion assay was performed using BD Biocoat invasion chambers coated with Matrigel™ (BD Biosciences, San Jose, CA) [21] according to manufacturer’s guidelines. Briefly, cells (2.5×10^4^/chamber) were incubated for 2 hr with medium with or without exosomes, seeded onto invasion chamber inserts, and incubated for 22 hr at 37°C. Non-invading cells were then removed, and invading cells were fixed with 100% methanol and stained with 0.5% crystal violet. The assays were performed in triplicates, six fields were counted per insert, and mean invasive cell numbers, fold changes and SDs were calculated.

### Statistical Analyses

All data were analyzed using ANOVA or Student’s *t* test, and represent the mean ± SD of at least triplicate samples. A *p* value less than 0.05 was considered statistically significant. Statistical tests were performed with SAS statistical software (version 9.1; SAS Institute, Cary, NC).

## Results

### Exosomes Identification and Characterization

**Figure 1:**
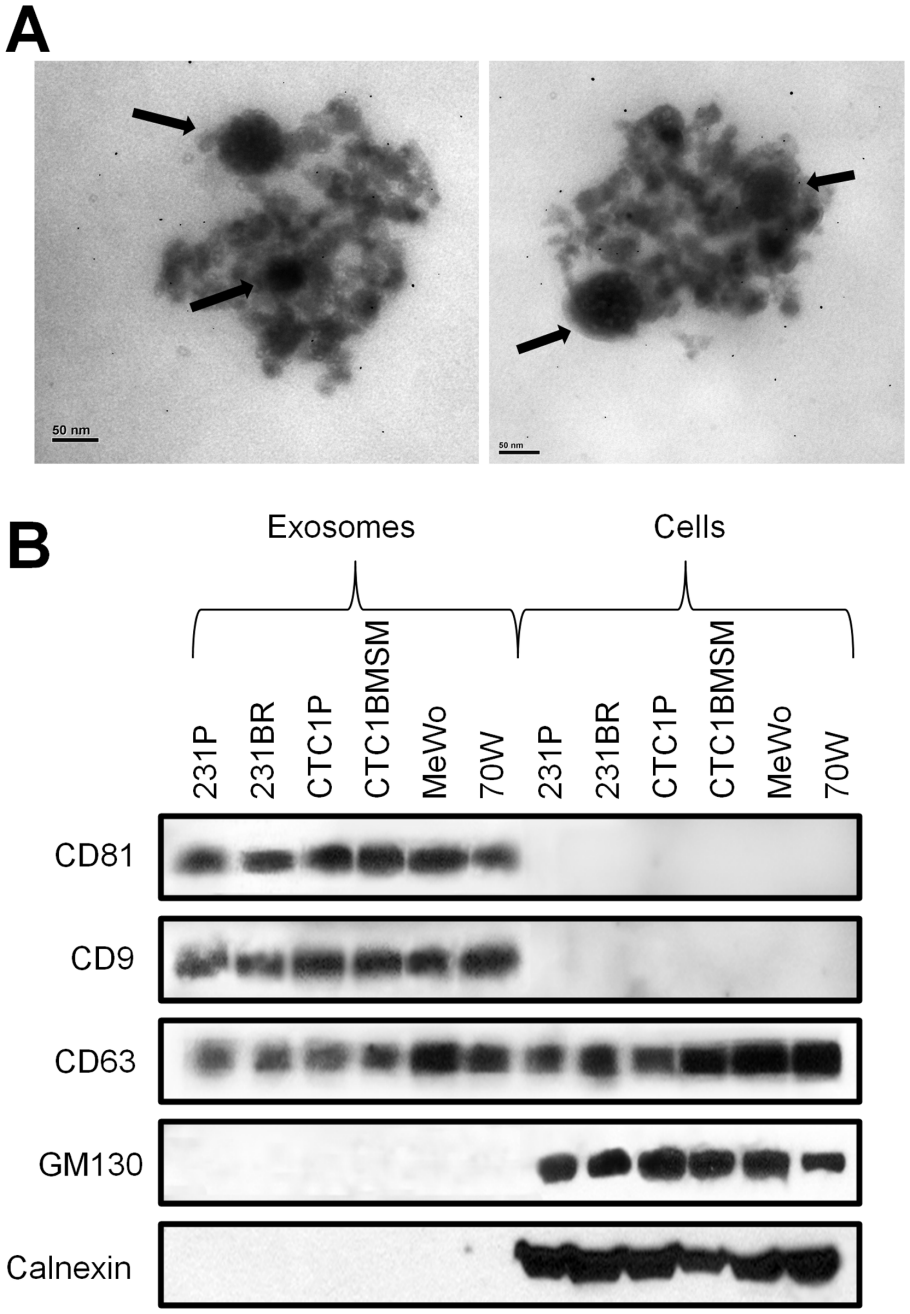
Identification and characterization of isolated exosomes. Exosomes were isolated by the ExoQuick-TC Precipitation Solution. (**A**) Representative morphological characterization of exosomes derived from brain metastatic 70 W melanoma cells by transmission electron microscopy. Round particles with characteristic exosomal size (30–100 nm) and shape were observed (arrows) immersed in the Exoquick solution. Scale bar is 50 nm. (**B**) Molecular confirmation of exosomes markers by Western blotting analysis. Exosome preparations were found to be positive for the exosomal markers CD9, CD63, and CD81 while negative for proteins from the endoplasmic reticulum (calnexin) and the Golgi apparatus (GM130) which were found to be present in cells lysates.

As initial step, we aimed to isolate exosome preparations from either parental human breast cancer (MDA-MB-231P, CTC1P) or melanoma (MeWo) cell lines, and corresponding brain metastatic variants selected from these cell lines (MDA-MB-231BR, CTC1BMSM, and 70 W, respectively) [19], [20], [21]. The selected method to isolate exosomes was ExoQuick-TC Precipitation Solution, a polymer based reagent. Next, transmission electron microscopy was employed to characterize the quality of vesicles. Round particles with a characteristic exosomal size (30–100 nm) and shape were observed immersed in the Exoquick solution (Fig. 1A) [2]. Exosomes were found to be positive for the exosomal markers CD9, CD63, and CD81, confirming these vesicles as exosomes (Fig. 1B) [22], [23]. CD9 and CD81 were enriched in exosomes compared to cells, as previously reported [24], [25]. Because other cell compartments can produce vesicles, the presence of proteins from the endoplasmic reticulum (calnexin) and the Golgi apparatus (GM130) was determined. These proteins were not found in exosomes while they were detected in cells (Fig. 1B), indicating that little or no contamination of vesicles from other cell compartments occurred in our exosome preparations.

### Differential miRNA Profiles of Exosomes from Brain Metastatic versus Non-brain Metastatic Cells

**Figure 2:**
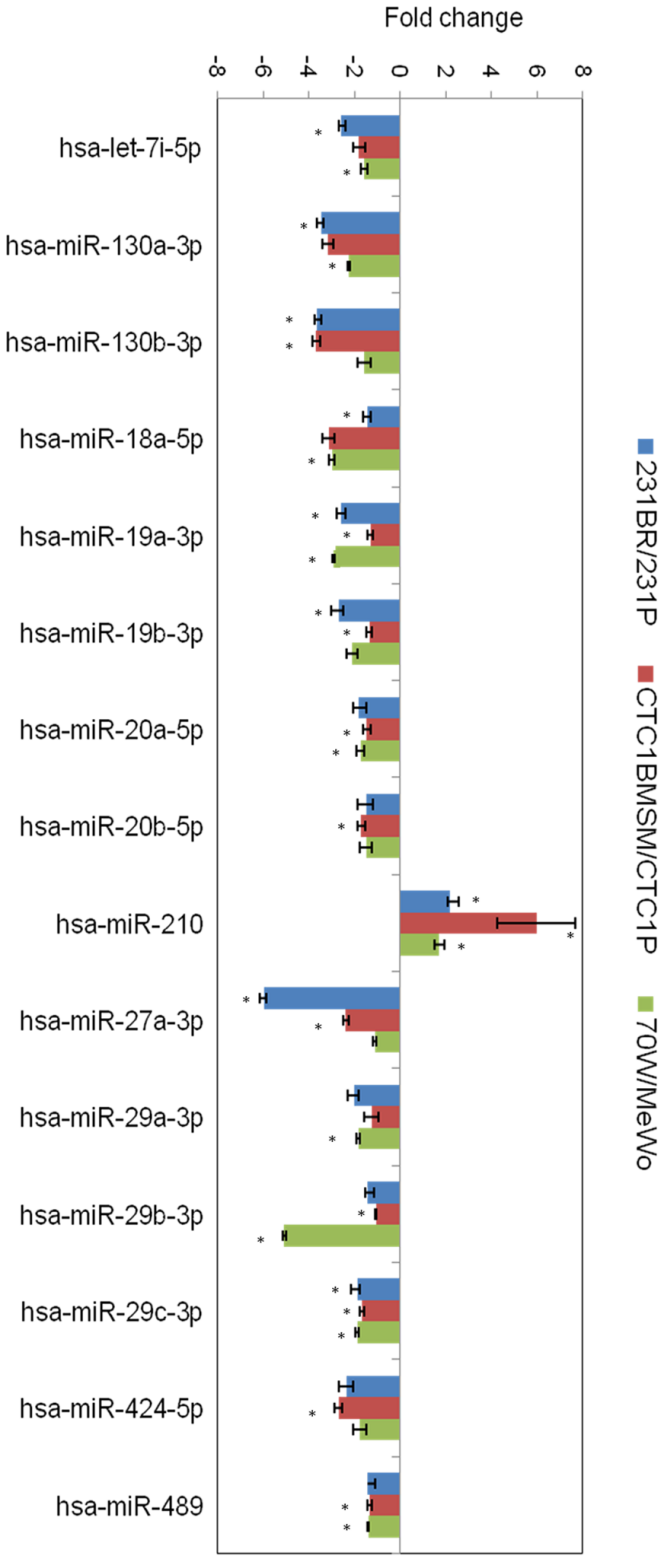
Differential miRNA profiles of exosomes from brain metastatic versus non-brain metastatic cells. Pathway-focused miScript miRNA PCR array was used to analyze the miRNAs contained within the exosomes. MiRNAs with statistically significant fold changes between brain metastatic (BM) and non-BM cell-derived exosomes were represented. Asterisks (*) denote statistically significant differences (p<0.05). MiR-210 was significantly enriched in all three BM cell-derived exosomes compared to non-BM, while two miRNAs were significantly down-regulated: miR-19a and miR-29c.

To determine exosomal miRNA content, we isolated the total RNA from exosomes derived from the six cell lines, and analyzed it by the Human Breast Cancer miScript miRNA Real-Time PCR Array. This technique profiles the expression of 84 miRNAs known or predicted to alter their expression during breast cancer initiation and/or progression. Among the 84 miRNAs, 60 were detected by the array in all exosomes (Fig. S1). Although the array is designed for breast cancer samples, miRNAs were equally detected in exosomes from melanoma cell lines. We calculated the fold change of miRNA expression between BM and non-BM cell-derived exosomes (231BR versus 231P, CTC1BMSM versus CTC1P, and 70 W versus MeWo), and we searched for similar patterns in the three groups. We identified one miRNA, miR-210, to be significantly enriched (p<0.05) in all three BM compared to non-BM cell-derived exosomes, while two miRNAs were significantly down-regulated (p<0.05), miR-19a and miR-29c (Fig. 2). Furthermore, twelve miRNAs were found to be significantly down-regulated (p<0.05) in at least one group and close to the significance level (p<0.15) in the other groups: let-7i, miR-130a, -130b, 27a, -424 and -489; along with four miRNAs belonging to the same family of miR-19a, the miR-17-92 family, and two belonging to the miR-29 family.

### Differential Protein Profiles of Cells versus Exosomes

**Figure 3:**
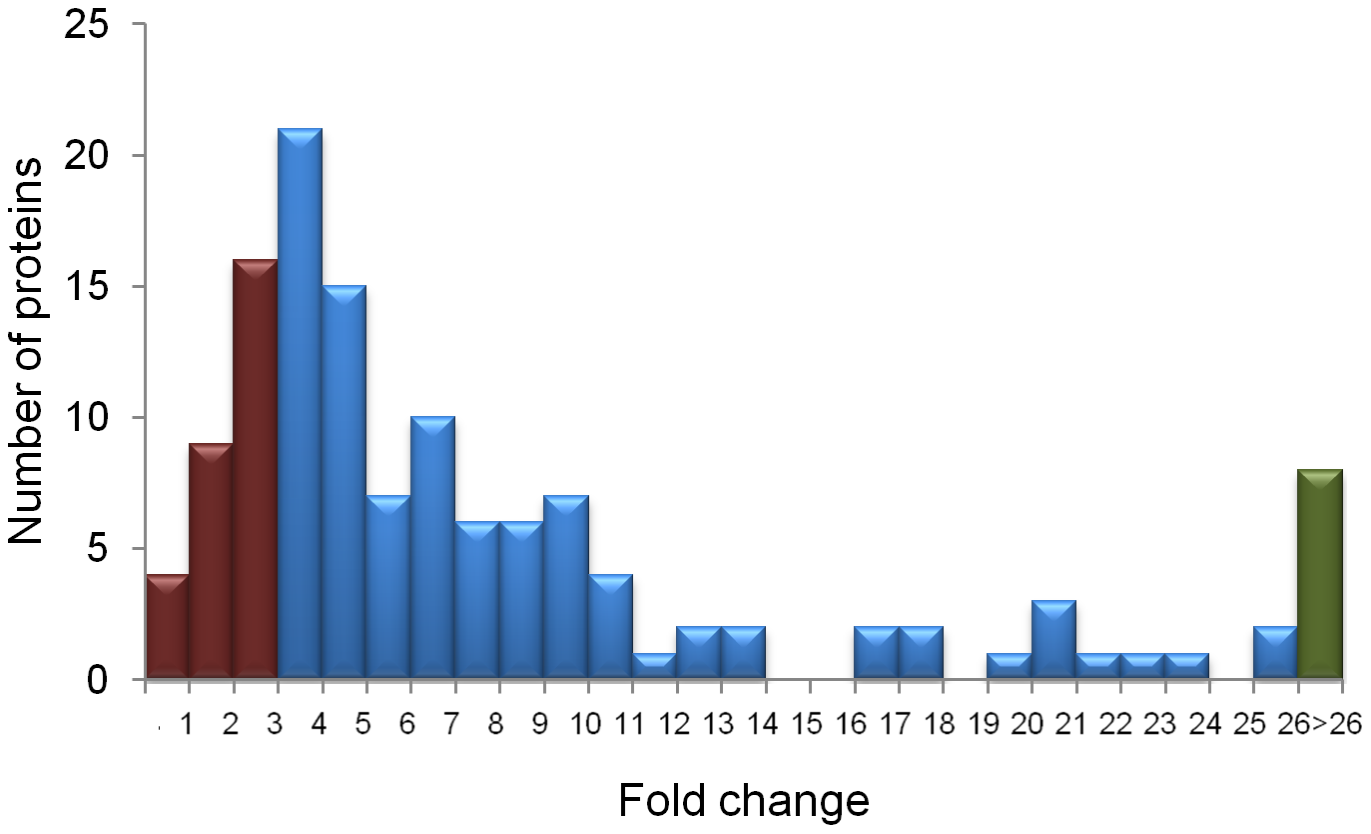
Differential protein profiles of exosomes compared to respective cells. Proteomic analyses were conducted using the Reverse Phase Protein Array by the RPPA Core Facility at MD Anderson Cancer Center (Houston, TX). Fold change of protein content in cells versus exosomes is represented by a histogram. The brown bars show the group of proteins that are present at high levels in exosomes compared to cells (0 to 3-fold change), the blue bars represent the bulk of the proteins (3 to 26-fold change), and the green bar shows the group of proteins detected at low quantities in exosomes (fold change higher than 26).

We aimed to investigate the protein content of our exosome preparations. To this end, we employed the reverse phase protein array (RPPA) technology, a useful platform for identifying dysregulated signaling pathways in tumors [26]. All 131 proteins included in the proteomic analyses were detected in the exosomes and are listed in Table S1. To identify proteins with a high content in exosomes in relation to cells, we calculated the fold change of protein content in cells versus exosomes, and represented it by a histogram (Fig. 3). Specifically, we focused on proteins whose fold change was between 0 and 3. We classified these proteins according to their cellular component, biological process, molecular function, and pathways (Fig. S2). Nucleus, cytosol, and plasma membrane were the cellular components where highly detected proteins in exosomes were mainly located, while proteins located in the extracellular space and organelles were found in fewer amounts. The biological processes in which these proteins were predominantly involved were cell communication, metabolic process, and cell cycle while their molecular function were mainly binding, catalytic activity, and receptor activity. Pathways in which most proteins were implicated were apoptosis, EGFR (epidermal growth factor receptor), cadherin, integrin, interleukin and Wnt signaling pathways. Among this group of proteins, only two (fibronectin and cyclin D1) were detected at higher levels in exosomes than in cells (0 to 0.7-fold change) while collagen VI, INPP4B (inositol polyphosphate-4-phosphatase) and N-Cadherin were expressed in cells and exosomes within the same range (0.7 to 1.3-fold change). We also categorized the group of proteins detected in a small quantity in exosomes compared to cells (fold change higher than 26), to identify proteins that are not an important cargo in the exosomes to be transported out of the cells. Among these eight proteins, we identified tumor suppressors such as caveolin1, Merlin/NF2 (neurofibromin 2), and tuberin.

### Differential Protein Profiles of Brain Metastatic versus Non-brain Metastatic Cells-derived Exosomes

**Figure 4:**
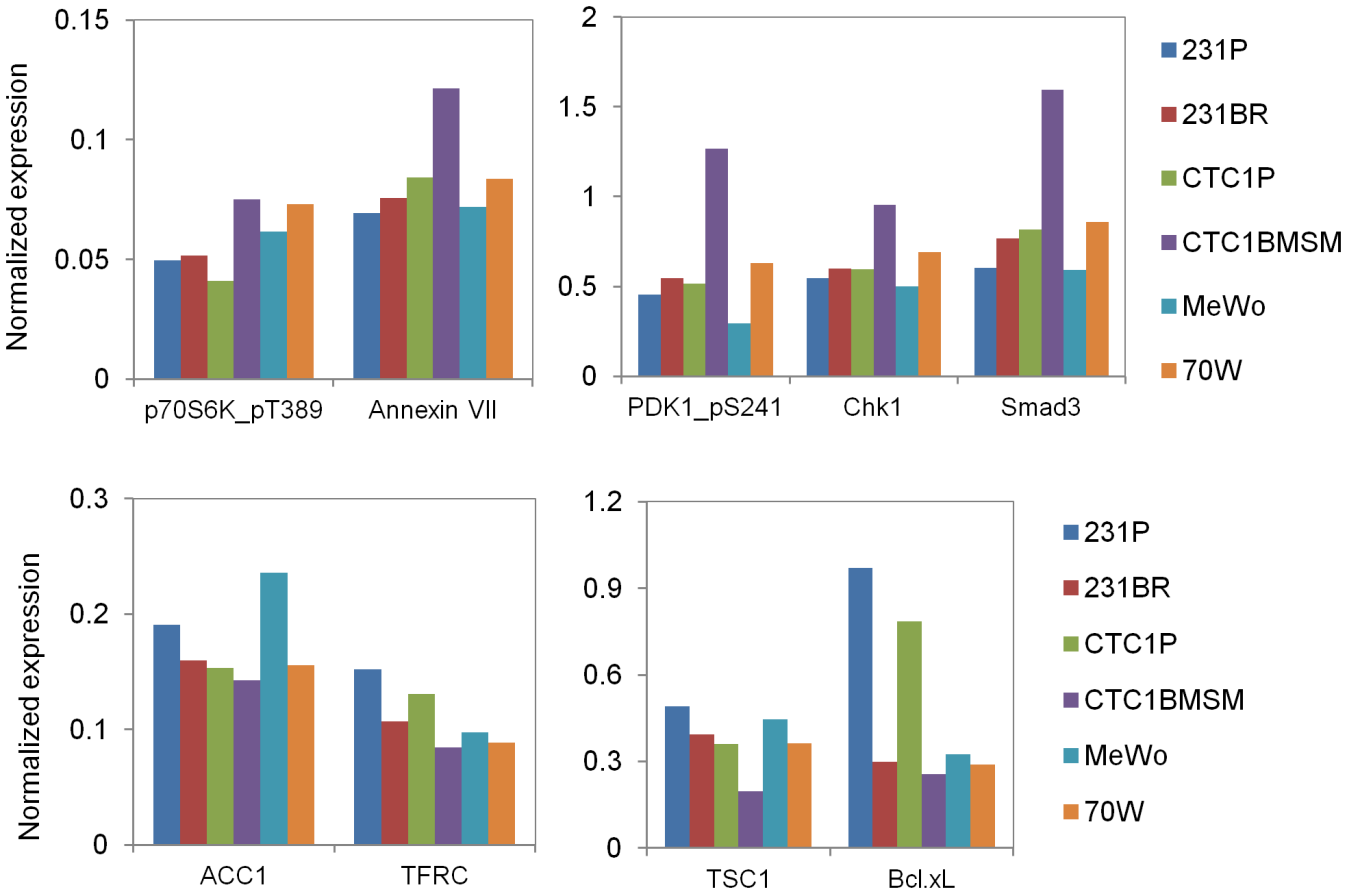
Differential protein profiles of brain metastatic versus non-brain metastatic cell-derived exosomes. The normalized expression of proteins detected in exosomes is represented according to the RPPA data. Five proteins up-regulated and four proteins down-regulated in the three BM exosomes compared with non-BM exosomes were identified. Of note, the highest expression among the up-regulated and the lowest expression among the down-regulated proteins occurred in the CTC1BMSM cell line in all cases.

Similarly to our miRNAs studies, we aimed to investigate similar protein patterns among exosomes derived from the three groups of cell lines (231BR versus 231P, CTC1BMSM versus CTC1P, and 70 W versus MeWo) (Fig. S3). We identified five proteins to be up-regulated (phospho-p70 S6 Kinase-Thr389, annexin VII, phospho-PDK1-Ser241, Chk1 and Smad3) while four proteins were down-regulated [ACC1 (acetyl CoA carboxylase), TFRC (transferrin receptor), TSC1 (tuberous sclerosis 1) and Bcl-x_L_ (B-cell lymphoma-extra large)] in the three BM exosomes compared with non-BM exosomes, although these differences were not highly significant (Fig. 4). Of interest, the higher expression among the up-regulated and the lower expression among the down-regulated proteins were detected in all cases in the CTC1BMSM cell line. This was also the case when analyzing the profile of many other proteins throughout the proteomic analysis (Fig. S3). Within the up-regulated group, slightest differences were observed between the 231P and 231BR cell lines.

### Cells Acquire Higher Adhesive and Invasive Capabilities by Uptaking Exosomes

**Figure 5:**
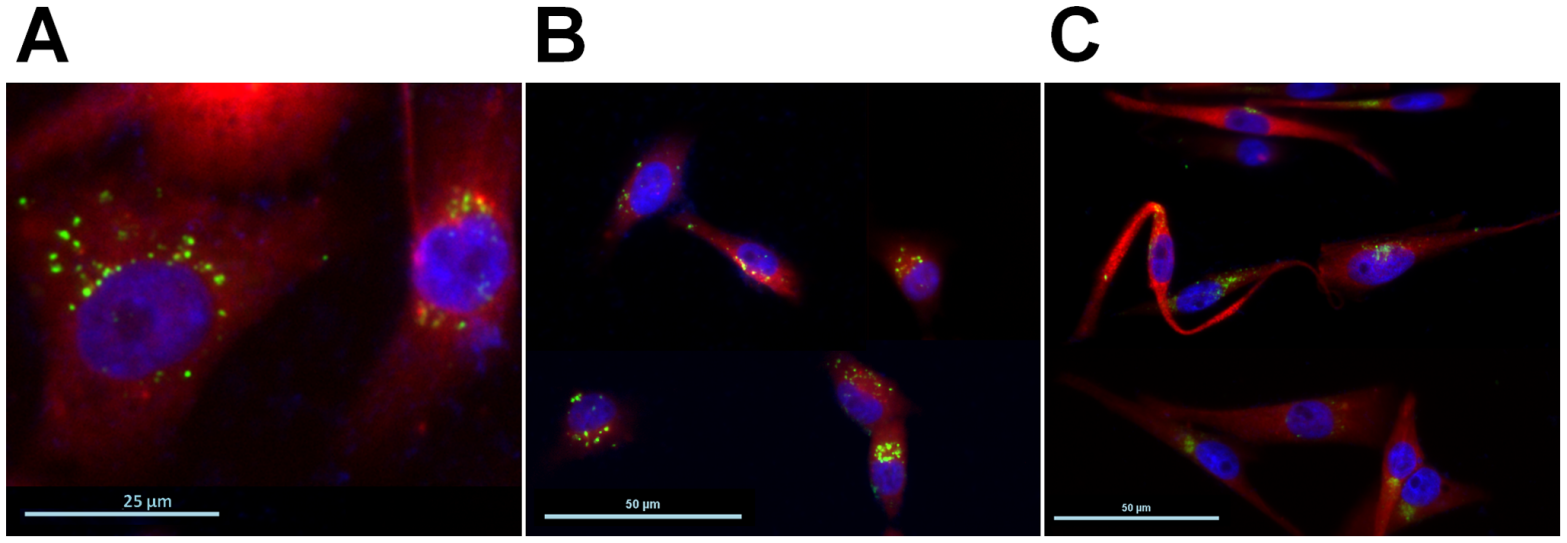
Non-brain metastatic cells uptake exosomes from their derivatives brain metastatic (BM) cell lines. Exosomes from the BM cell lines were labeled with green lipophilic fluorescent dye PKH67 and incubated for 5-BM cells transduced with Tubulin-RFP and visualized by fluorescence microscopy. (**A**) MDA-MB-231P (231P) cells with MDA-MB-231BR-derived exosomes. (**B**) CTC1P cells with CTC1BMSM-derived exosomes. (**C**) MeWo cells with 70 W-derived exosomes. Numerous green fluorescent labeled exosomes were observed inside the cells, mostly located at the perinuclear region.

To test whether exosomes derived from BM cells could be internalized by non-BM cells, and transport and deliver their cargo for subsequent effects on cell behavior, we incubated exosomes labeled with the green lipophilic fluorescent dye PKH67 with cells transduced with Tubulin-RFP. Numerous labeled particles were observed inside the cells by fluorescence microscopy, and they were mainly located at the perinuclear region, showing that all non-BM cell lines were able to uptake the exosomes from their BM variants (Fig. 5). We hypothesized that once the cargo is released, it might affect the metastatic capabilities of cells; therefore, we analyzed the proliferative, adhesive, and invasive potential of cells following exosomes internalization. We tested the variation in cell proliferative capabilities following the addition of their own exosomes or exosomes from their derivatives. We did not observe any significant differences among the two groups in any of the cell lines (Fig. S4 represents the 48 hr assay).

**Figure 6:**
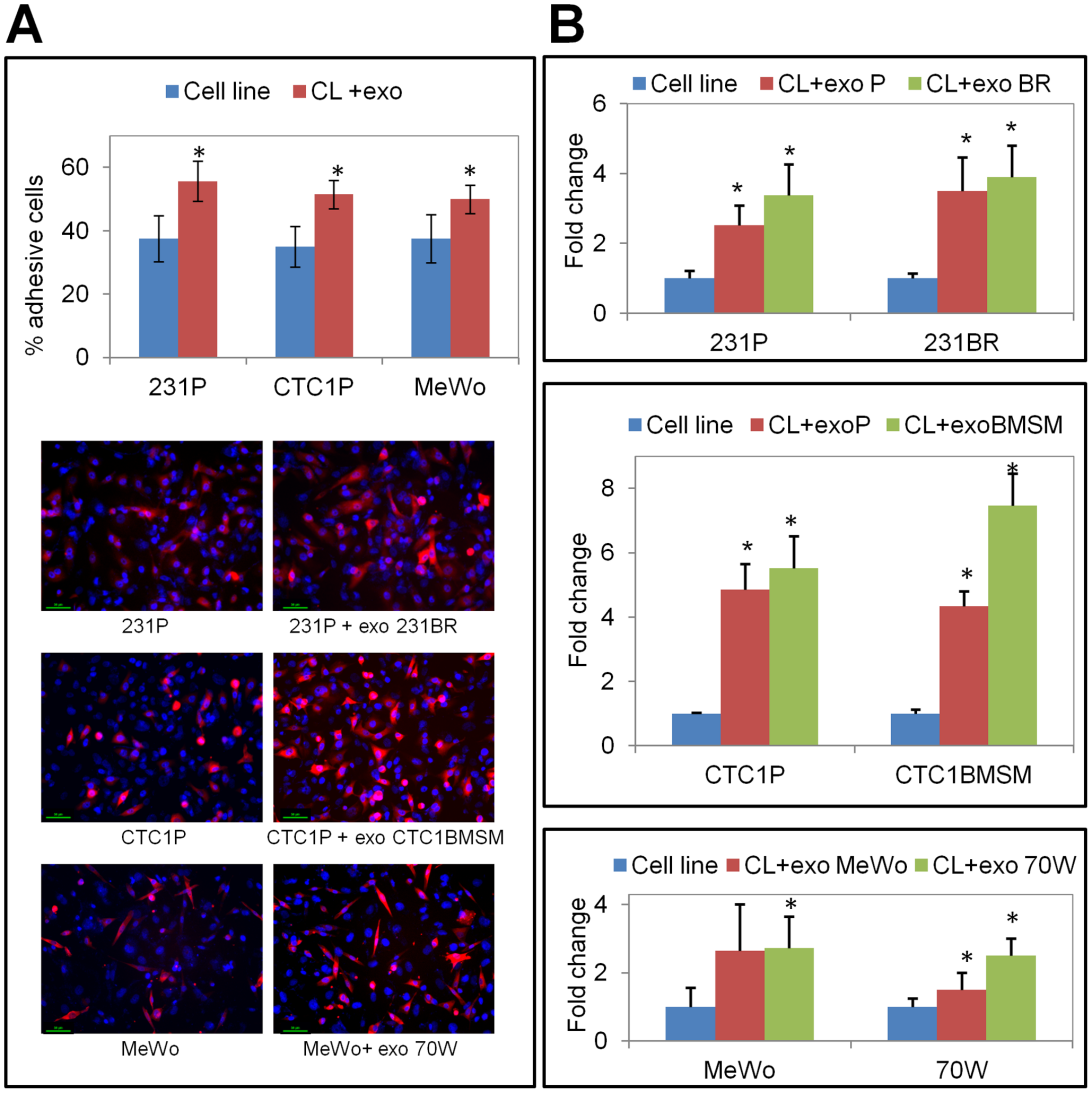
Cells acquire a higher adhesive and invasive potential through uptaking exosomes. (**A**) Tubulin-RFP transduced non-BM cell lines with and without exosomes from BM cells were plated over a human brain microvascular endothelial cells (HBMEC) monolayer. All non-BM cell lines increased their adhesive potential when exosomes are added compared to cells without exosomes. (**B**) Cells without exosomes, cells incubated with their own exosomes (parental cell line-derived exosomes), and cells incubated with the exosomes from their cell variants (BM cell line-derived exosomes) were plated onto invasion chambers coated with Matrigel™ artificial basement membrane. Cells incubated with exosomes showed a higher invasive capability compared to the cells without exosomes. Asterisks (*) denote statistically significant differences (p<0.05).

To analyze the adhesive potential of the cells to the brain endothelium, we plated non-BM cell lines over a HBMEC cells monolayer to reflect a scenario resembling more closely to the one occurring *in vivo*. To differentiate HBMEC from non-BM cells, we transduced the latter with Tubulin-RFP, and compared the number of adherent cells plated with and without exosomes from BM cells. Interestingly, all non-BM cell lines significantly (p<0.05) increased their adhesive potential by approximately 20% when exosomes were added compared to cells without exosome addition (Fig. 6A).

To examine tumor cell invasiveness, we incubated cells with either their own exosomes or with exosomes from their respective cell variants, and plated the cells with or without exosomes on invasion chambers coated with the artificial basement membrane Matrigel™. We found that cells incubated with exosomes, regardless of their origin, had a higher invasive capability compared to cells alone. This increase corresponds to a 2 to 7-fold change, and was found to be statistically significant in all cases, except for MeWo cells when they were incubated with their own exosomes (Fig. 6B).

## Discussion

In this study, we investigated the differential microRNA and protein cargo of exosomes isolated from brain-colonizing breast cancer and melanoma cell lines, and how this cargo can affect the brain invasive properties and metastatic potential of these cells. We identified dysregulated miRNAs and proteins in BM versus non-BM cell-derived exosomes and found an increase in adhesion and invasion properties in non-BM cells when they are incubated with BM cell-derived exosomes.

Tumor cells can establish a suitable microenvironment (pre-metastatic niche) for metastasizing cells [27]. Recent studies support the abilities of tumor cell-derived exosomes to modulate the surrounding microenvironment to make it more permissive for tumor invasion and growth [7]. Tumor exosomes are known to carry proteins, mRNAs, and miRNAs that can play key roles in these processes. The expression profiling of exosomal miRNAs has been shown to be significantly different among lung [16] or ovarian [9] cancer patients (among others) compared to healthy controls. MiRNAs have been proposed to contribute to oncogenesis by functioning either as tumor suppressors or oncogenes [28]. Our exosome miRNA profiling report revealed similarities between the three groups of cell lines. By deciphering miRNAs that were significantly up-regulated (p<0.05) in all three BM cell-derived exosomes, we detected a 2 to 6-fold increase of miR-210 expression. This miRNA has been described to be induced in response to hypoxia [29] with its expression known to be elevated in multiple cancer types [30], [31], and correlating with breast and melanoma metastasis [32], [33]. A few studies have identified miR-210 in exosomes derived from ovarian or lung cancer, but a correlation between this miRNA levels in exosomes and the stage of disease could not be assessed [9], [16]. According to our results, exosomal miR-210 could be considered as an independent prognostic factor in brain metastatic breast cancer and melanomabecause the single miR-210 assay has been proposed to be prognostic factor in breast cancer patients [34]. Further, two miRNAs were significantly down-regulated in all three BM exosomes, miR-19a and miR-29c. MiR-19a belongs to the miR-17-92 family, along with miR-18a, -19b, 20a and -20b, all of them found down-regulated in BM exosomes. These microRNAs are considered oncogenes, promoting proliferation and tumor angiogenesis [35], [36], and their up-regulation has been observed in human cancer cells, including breast cancer [37]. Conversely, miR-17-92 family was found to be deleted in approximately 20% of ovarian, breast cancers and melanomas [38], and it was expressed at higher levels in non-metastatic MCF-7 compared with metastatic MDA-MB-231 cells, along with an inhibition of cellular invasion and tumor metastasis through cyclin D1 repression [39]. Therefore, both oncogenic and anti-tumorigenic capabilities of these miRNAs are not mutually exclusive as their functions are dictated by the targets expressed in their specific environment. MiR-29c has been found to be down-regulated in nasopharyngeal carcinomas, and up-regulating mRNAs encoding extracellular matrix proteins that are involved in metastasis [40], and found to negatively correlate with lung cancer brain metastasis [41]. However, there are no studies detecting a differential expression of miR-29c in tumor-derived exosomes. The other two members of the miR-29 family, miR-29a and -29b, were also down-regulated in BM exosomes. Our results provide the initial evidence of roles of these families as potential biomarkers; however, further studies are necessary to explore these miRNA families as prognostic factors in brain metastatic cancers.

To elucidate which proteins could be of relevance among the exosomes cargo as they can affect the metastatic potential of the cells, we performed a proteomic analysis in cell lines and derived exosomes. By analyzing protein expression in cells compared to exosomes, we found fold changes between 0.09 and 198, with an average 10.5-fold change for all proteins. We selected the group of proteins with low fold change (0 to 3) in cells versus exosomes to identify the proteins that are present at high levels in exosomes in relation to their cellular levels (Fig. 3). Several of these proteins have been identified previously in exosomes and some are novel. We classified these proteins according to their gene ontology. Surprisingly, we found the nucleus as the most common cellular component for exosomal proteins by detecting high levels of nuclear proteins, e.g. androgen and estrogen receptors (AR and ERα) or c-myc, a nuclear transcription factor and important regulator of cell growth playing multiple roles in breast cancer development and progression [42]. The main biological process in which these proteins were involved was cell communication which is the key role of exosomes. For example, among this group were PDK1, 14-3-3ε or receptors such as AR, ERα and HER3 (human epidermal growth factor receptor 3), with many roles in cell-cell signaling and signal transduction. Further, many other proteins in this group are known to be implicated in cell cycle regulation, e.g. cyclin D1, MAPK (mitogen-activated protein kinase), Akt, p27 and Src. Binding, catalytic and receptor activities were the predominant molecular functions (Fig. S2). Finally, proteins detected at high levels in exosomes were organized by pathways in which they participate. The pathways with more proteins implicated were apoptosis, EGFR, cadherin, integrin, interleukin and Wnt signaling, all of them keenly involved in tumorigenesis and metastasis.

Fibronectin was the protein detected at highest quantity in exosomes of our study. This protein may play a major role within the exosomes cargo transported to distant sites, since it is involved in the adhesion of many cell types [43], and it mediates both invasion and metastasis in melanoma and breast cancer cells [44], [45]. Furthermore, a specific up-regulation of fibronectin at the pre-metastatic niche before tumor cell arrival was proven to be indispensable for the initial stages of metastasis [27]. The second protein detected at a higher amount in exosomes than in cells was cyclin D1 which is encoded by a well-established human oncogene that is considered to be an important regulator of cell cycle progression. Its over-expression has been linked to the development and progression of breast cancer and melanomas among others [46], [47]. Accordingly, this protein could have important roles in the ability of breast cancer and melanoma-derived exosomes to alter their microenvironment, aiding to tumor cell survival and metastasis. Conversely, the group of proteins detected in low quantities in exosomes compared to cells included important tumor suppressors such as NF2 [48] and tuberin [49]. These results may provide clues for specific mechanisms by which cells sort proteins to be included in the exosome cargo.

By profiling dysregulated proteins between BM and non-BM cell-derived exosomes of the three groups, we detected up-regulated kinases involved in cell cycle control (Chk1) [50] and pathways controlling cell growth, proliferation and survival (Phospho-p70 S6 Kinase and Phospho-PDK1) [51]; Smad3, a protein that functions as a transcriptional modulator activated by transforming growth factor-beta [52], and annexin VII, that has been shown to correlate with metastatic breast cancer [53]. The up-regulation of these proteins, especially in circulating tumor cells, should be further studied in relation to the brain metastasis onset. Conversely, we identified a group of proteins that were down-regulated in BM exosomes: ACC1, TFRC, TSC1 and Bcl-x_L_. The differences between parental and brain-colonizing cell line-derived exosomes were not very dramatic in the first three proteins; however, Bcl-x_L_ was specifically up-regulated in exosomes from the non-BM breast cancer cell lines.This is in agreement with previous findings associating over-expression of this anti-apoptotic protein with nodal metastasis but not brain metastasis in human breast cancer tumors [54], [55].

An important finding from our study is that BM tumor cell-derived exosomes were internalized by non-BM cells. We interrogated whether the proteins and miRNAs contained within the exosome cargo could modify the metastatic potential of the cells following exosome uptake by altering intra and extravasation processes involving tumor cell adhesion and transmigration through the endothelium and underlying basement membranes. To this end, we analyzed whether the melanoma and breast cancer cells increase capabilities to adhere to human brain microvascular endothelial cells upon incubation with exosomes derived from BM cell lines. We detected a significant increase in all cell lines considered. Furthermore, all cells significantly increased their invasive capabilities upon incubation with exosomes, either homotypic or heterotypic cell variants-derived. These results suggest that BM cell-derived exosomes content possess distinctive proteins with key roles altering breast cancer and melanoma progression. We did not observe an increase in tumor cell proliferation following exosomes addition which could be ascribed to pro-apoptotic functions of tumor-derived exosomes. Exosomes isolated from the sera of oral or ovarian cancer patients and pancreatic tumor cells-derived exosomes have been shown to inhibit proliferation and induce apoptosis of T lymphocytes [56], [57]. In addition to immunosuppressive properties, a pro-apoptotic function of tumor-derived exosomes directly on tumor cells was also reported in pancreatic cancer [58]. These findings are in agreement with our results since the apoptosis signaling pathway was the one with most proteins implicated, e.g., caspase 7, a member of the caspase family, has been shown to be an effector protein of apoptosis. Therefore, these functions would counteract the proliferative functions for a portion of the proteins present in the exosomes cargo.

In summary, our investigations represent the first comprehensive analysis of microRNA and protein profiling of brain metastatic tumor cell-derived exosomes, and can be considered the initial and important step for further investigations to implicate exosome as clinically useful tools to provide prognostic value and new therapeutic directions in BM disease.

## Supporting Information

Figure S1 **MicroRNA analyses in exosomes from brain metastatic (BM) and non-BM cell lines.** Fold change of miRNA expression between brain metastatic (BM) and non-BM cell-derived exosomes (MDA-MB-231BR versus MDA-MB-231P, CTC1BMSM versus CTC1P and 70W versus MeWo) was calculated and represented on a heatmap. Pathway-focused miScript miRNA PCR array was used to analyze the miRNAs content in the exosomes. Sixty miRNAs among the 84 were detectable by the array in all exosomes. Asterisks (*) denote statistically significant differences (p<0.05).
(TIF)

Figure S2 **Classification of the proteins with a 0 to 3-fold change according to the gene ontology.** Classification was done by the Protein Analysis Through Evolutionary Relationships Classification System (http://www.pantherdb.org). Nucleus, cytosol and plasma membrane were the cellular components where the proteins highly detected in the exosomes were mainly located. The biological processes in which these proteins were principally involved were cell communication, metabolic process and cell cycle and their molecular function were predominantly binding, catalytic activity and receptor activity. The pathways in which most proteins were implicated were apoptosis, EGFR, cadherin, integrin, interleukin and Wnt signaling pathways.
(TIF)

Figure S3 **Differential protein profiles of brain metastatic versus non-brain metastatic cell-derived exosomes.** Normalized expression of the proteins detected in the exosomes by RPPA analysis is represented by heatmap.
(TIF)

Figure S4 **Tumor cells do not acquire a higher proliferative potential through uptaking exosomes.** The proliferative capability of cells was measured by the MTT assay. Non-BM cell lines were seeded on a 96-well plate and incubated overnight (16 hr). Cells were then incubated with or without exosomes, and MTT was added after 48 h. No statistically significant differences were found among the groups in any of the cell lines considered.
(TIF)

Table S1 **Differentially identified protein fold change between cells and exosomes.** Proteomic analyses were conducted using the Reverse Phase Protein Array by the RPPA Core Facility at MD Anderson Cancer Center (Houston, TX). Fold change of protein content in cells versus exosomes was calculated. Brown color shows the group of proteins that are present at high levels in exosomes compared to cells (0 to 3-fold change), blue color represents the bulk of the proteins (3 to 26-fold change), and green color shows the group of proteins detected at low quantities in exosomes (fold change higher than 26).
(DOCX)

Protocol S1 **RPPA methodology.** Methodology employed by the RPPA Core Facility at MD Anderson Cancer Center (Houston, TX) to perform the Reverse Phase Protein Array.
(DOCX)
