# Supplementary figures and images for: MicroRNA and Protein Profiling of Brain Metastasis Competent Cell-Derived Exosomes
Source: PLoS One. 2013 Sep 16;8(9):e73790. doi: 10.1371/journal.pone.0073790 (PMC3774795; doi:10.1371/journal.pone.0073790)

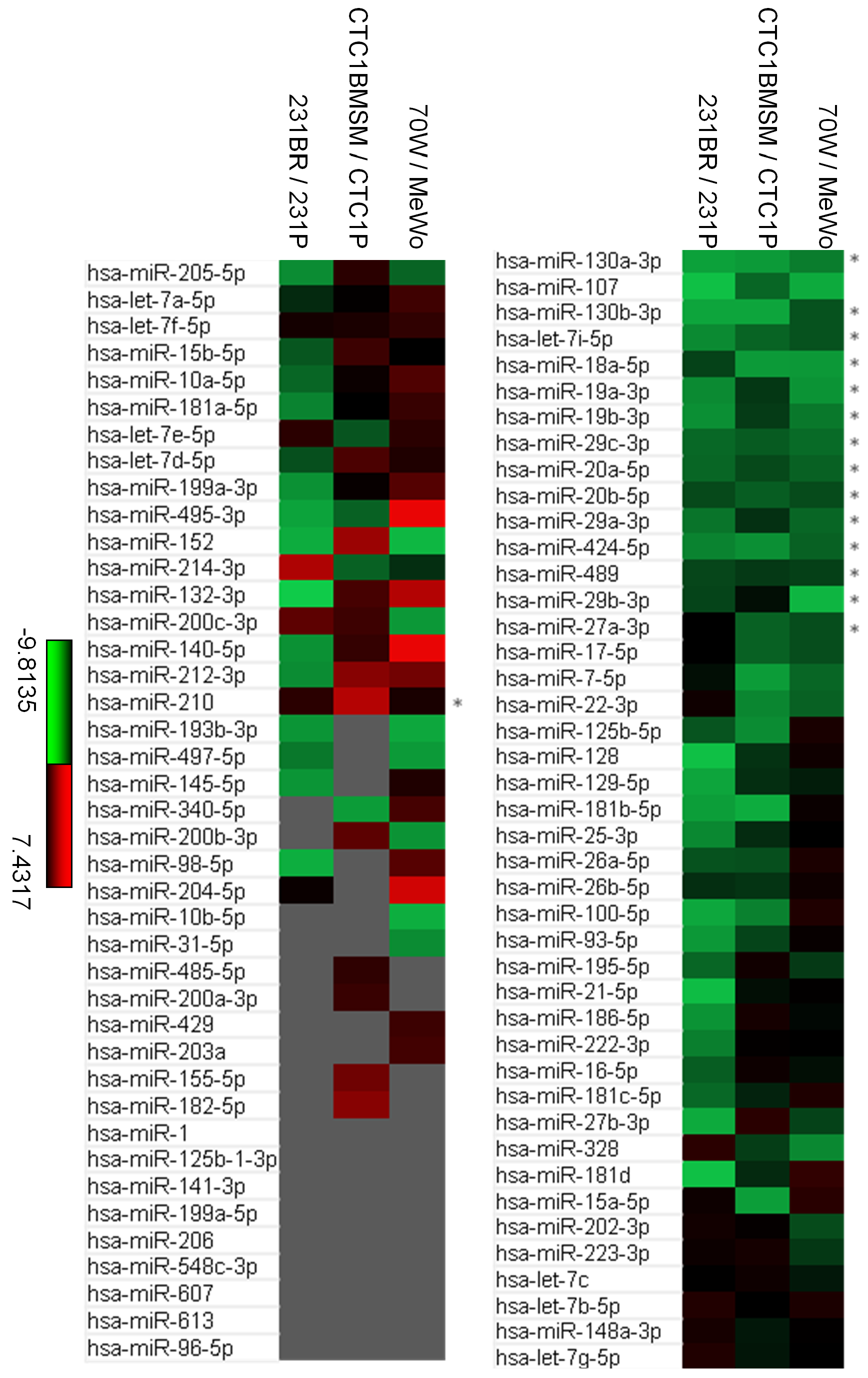

Supplement: Figure S1 — MicroRNA analyses in exosomes from brain metastatic (BM) and non-BM cell lines. Fold change of miRNA expression between brain metastatic (BM) and non-BM cell-derived exosomes (MDA-MB-231BR versus MDA-MB-231P, CTC1BMSM versus CTC1P and 70W versus MeWo) was calculated and represented on a heatmap. Pathway-focused miScript miRNA PCR array was used to analyze the miRNAs content in the exosomes. Sixty miRNAs among the 84 were detectable by the array in all exosomes. Asterisks (*) denote statistically significant differences (p<0.05). (TIF) [file pone.0073790.s001.tif]

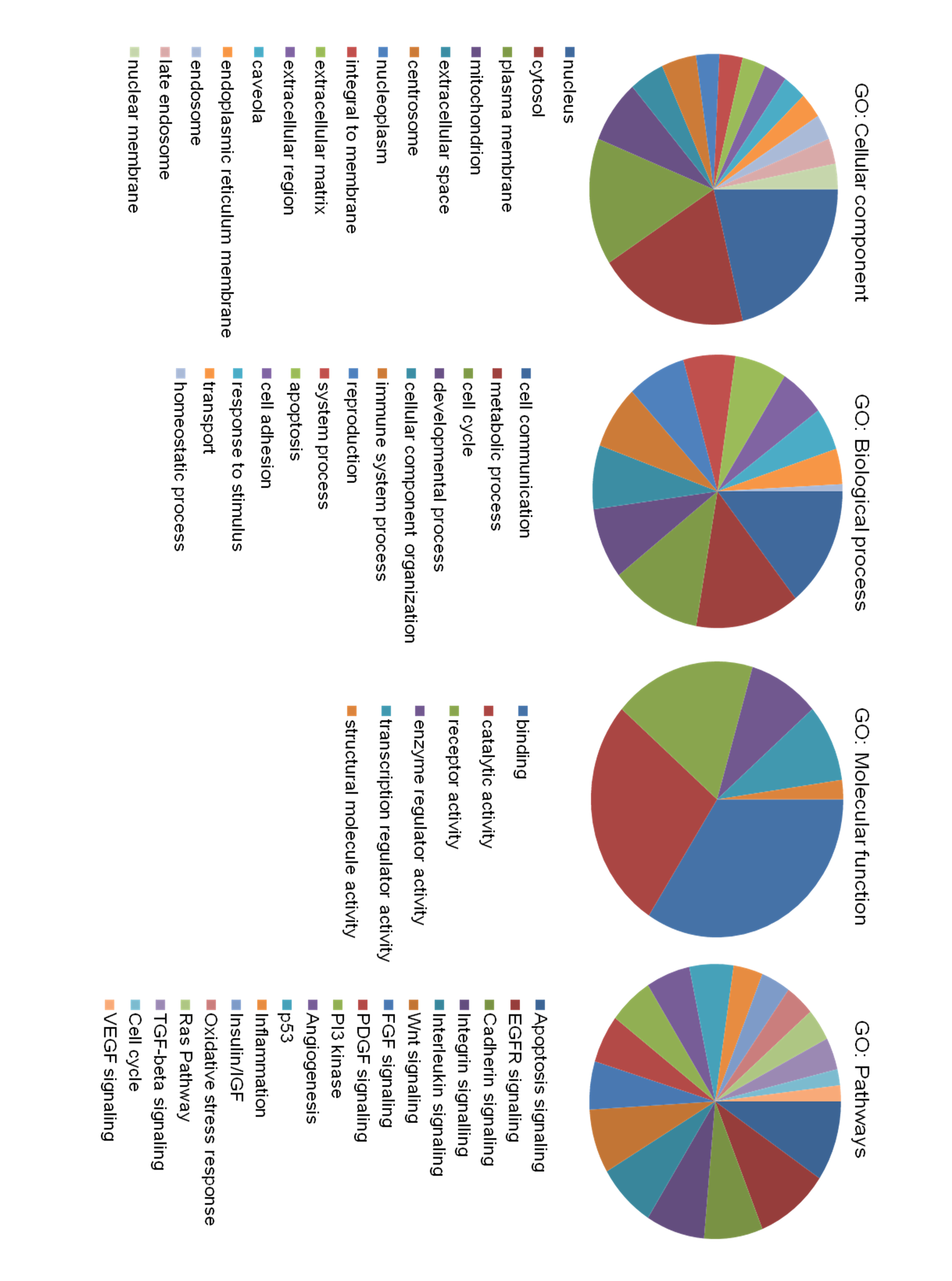

Supplement: Figure S2 — Classification of the proteins with a 0 to 3-fold change according to the gene ontology. Classification was done by the Protein Analysis Through Evolutionary Relationships Classification System (http://www.pantherdb.org). Nucleus, cytosol and plasma membrane were the cellular components where the proteins highly detected in the exosomes were mainly located. The biological processes in which these proteins were principally involved were cell communication, metabolic process and cell cycle and their molecular function were predominantly binding, catalytic activity and receptor activity. The pathways in which most proteins were implicated were apoptosis, EGFR, cadherin, integrin, interleukin and Wnt signaling pathways. (TIF) [file pone.0073790.s002.tif]

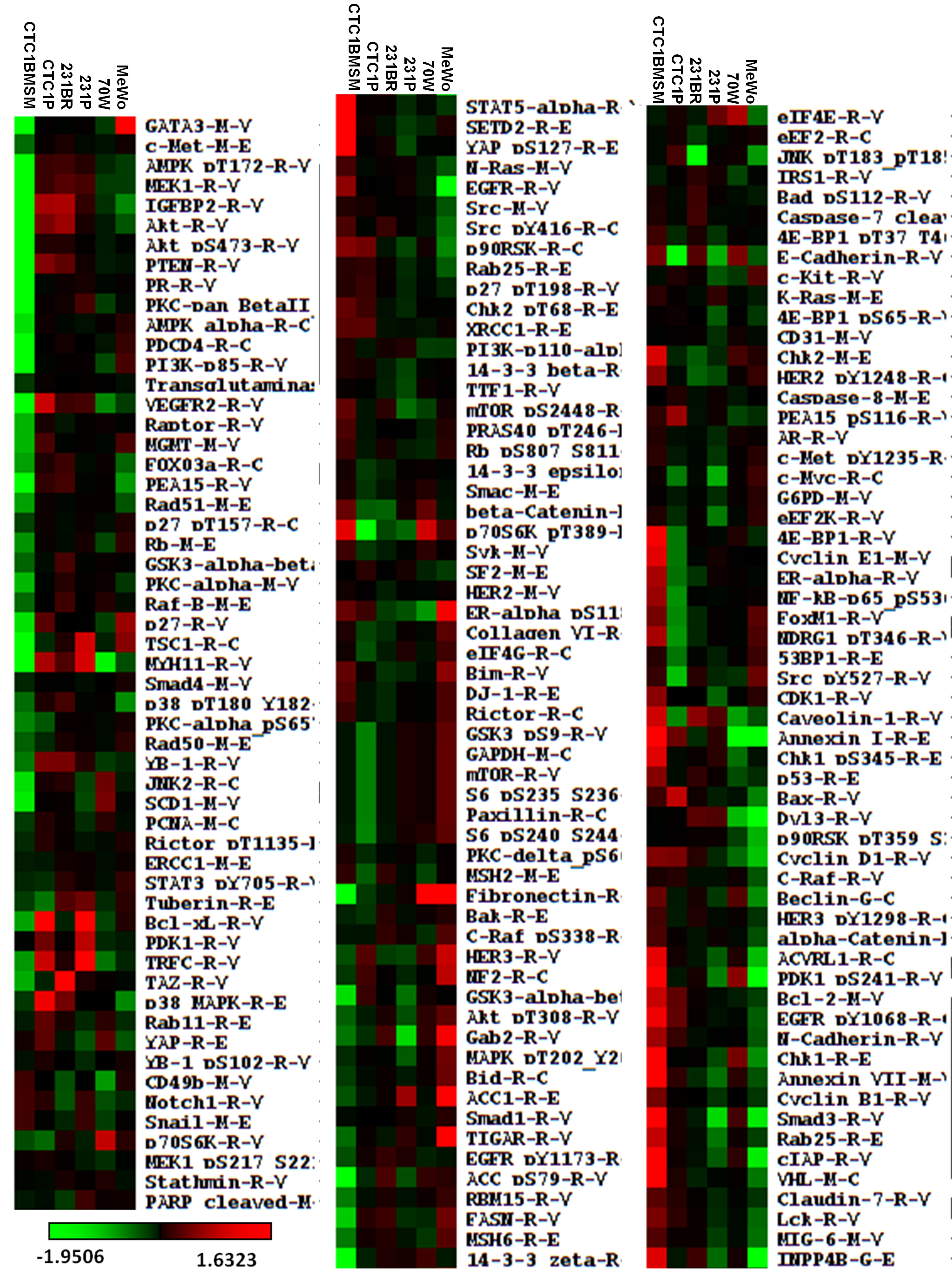

Supplement: Figure S3 — Differential protein profiles of brain metastatic versus non-brain metastatic cell-derived exosomes. Normalized expression of the proteins detected in the exosomes by RPPA analysis is represented by heatmap. (TIF) [file pone.0073790.s003.tif]

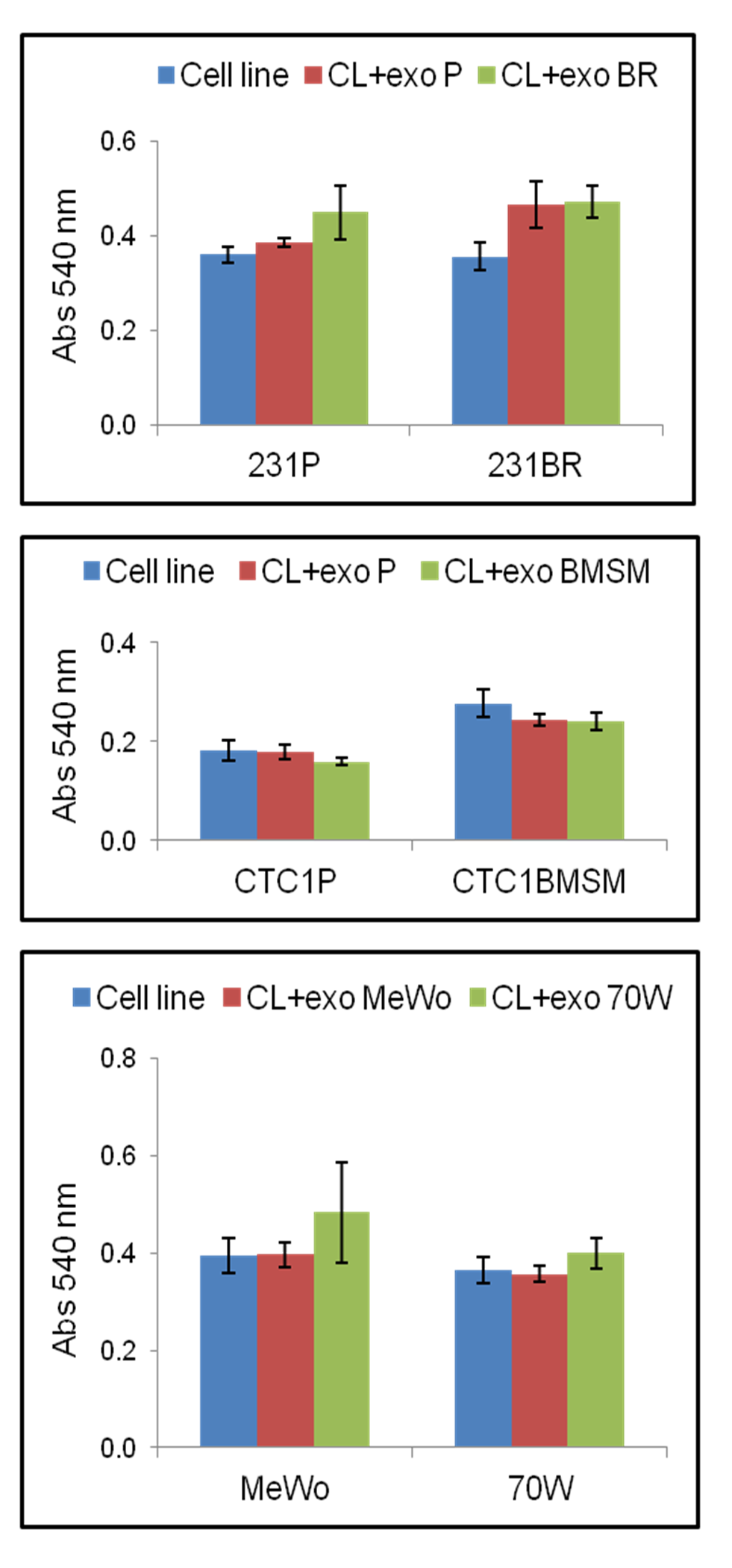

Supplement: Figure S4 — Tumor cells do not acquire a higher proliferative potential through uptaking exosomes. The proliferative capability of cells was measured by the MTT assay. Non-BM cell lines were seeded on a 96-well plate and incubated overnight (16 hr). Cells were then incubated with or without exosomes, and MTT was added after 48 h. No statistically significant differences were found among the groups in any of the cell lines considered. (TIF) [file pone.0073790.s004.tif]
