# Supplementary material for: MicroRNA and Protein Profiling of Brain Metastasis Competent Cell-Derived Exosomes
Source: PLoS One. 2013 Sep 16;8(9):e73790. doi: 10.1371/journal.pone.0073790 (PMC3774795; doi:10.1371/journal.pone.0073790)
Supplement: Table S1 — Differentially identified protein fold change between cells and exosomes. Proteomic analyses were conducted using the Reverse Phase Protein Array by the RPPA Core Facility at MD Anderson Cancer Center (Houston, TX). Fold change of protein content in cells versus exosomes was calculated. Brown color shows the group of proteins that are present at high levels in exosomes compared to cells (0 to 3-fold change), blue color represents the bulk of the proteins (3 to 26-fold change), and green color shows the group of proteins detected at low quantities in exosomes (fold change higher than 26). (DOCX) [file pone.0073790.s005.docx]

| 0 to 3-fold change | | | | | | | 3 to 26-fold change | | | | | | | | | | |
| --- | --- | --- | --- | --- | --- | --- | --- | --- | --- | --- | --- | --- | --- | --- | --- | --- | --- |
| Fibronectin | 0.09 | | EGFR_pY1173 | | 2.18 | | Rad51 | | 3.08 | | Rb | | 3.84 | p90RSK_pT359_S363 | | | 4.62 |
| Cyclin_D1 | 0.63 | | ERCC1 | | 2.22 | | MEK1_pS217_S221 | | 3.09 | | STAT3_pY705 | | 3.86 | AMPK_alpha | | | 4.67 |
| Collagen_VI | 0.99 | | Caspase7 | | 2.25 | | Bim | | 3.14 | | PARP_cleaved | | 3.91 | ER.α_pS118 | | | 4.69 |
| INPP4B | 0.99 | | X14.3.3_epsilon | | 2.25 | | CDK1 | | 3.14 | | Bcl.2 | | 3.93 | Notch1 | | | 4.96 |
| N.Cadherin | 1.29 | | Chk2_pT68 | | 2.29 | | Stathmin | | 3.16 | | Smad3 | | 3.94 | p53 | | | 5.15 |
| PDK1_pS241 | 1.51 | | p27_pT157 | | 2.30 | | c.Kit | | 3.17 | | TAZ | | 4.01 | AMPK_pT172 | | | 5.19 |
| AR | 1.55 | | JNK_pT183_pT185 | | 2.43 | | MIG.6+ | | 3.22 | | Caspase.8 | | 4.12 | Chk1_pS345 | | | 5.36 |
| Akt_pT308 | 1.59 | | Claudin.7 | | 2.54 | | c.Met_pY1235 | | 3.35 | | XRCC1 | | 4.19 | PCNA | | | 5.44 |
| Beclin | 1.65 | | HER3_pY1298 | | 2.55 | | Smad1 | | 3.44 | | Snail | | 4.29 | IGFBP2 | | | 5.69 |
| c.Myc | 1.67 | | c.Met | | 2.71 | | Rab11 | | 3.51 | | p27 | | 4.29 | HER2_pY1248 | | | 5.82 |
| Erα | 1.74 | | CD31 | | 2.78 | | p38_pT180_Y182 | | 3.53 | | Cyclin_E1 | | 4.29 | Bax | | | 5.93 |
| Rab25 | 1.92 | | Bcl.xL | | 2.87 | | p27_pT198 | | 3.54 | | C.Raf | | 4.33 | Rad50 | | | 6.12 |
| Bid | 2.00 | | IRS1 | | 2.94 | | PR | | 3.56 | | Dvl3 | | 4.37 | Bak | | | 6.13 |
| Chk1 | 2.12 | | Smad4 | | 2.97 | | GATA3 | | 3.60 | | YB.1_pS102 | | 4.37 | FOX03a | | | 6.27 |
| Src | 2.14 | |  | |  | | C.Raf_pS338 | | 3.69 | | EGFR_pY1068 | | 4.45 | YAP | | | 6.43 |
|  |  | |  | |  | | Src_pY416 | | 3.81 | | Syk | | 4.57 | S6_pS235_S236 | | | 6.50 |
| 3 to 26-fold change | | | | | | | | | | | | >26-fold change | | | |  |  |
| Akt_pS473 | | 6.63 | | Annexin_VII | | 8.92 | | GSK3.α.β._pS21_S9 | | 13.42 | | Tuberin | | | 32.67 |  |  |
| PKC.α | | 6.66 | | p70S6K_pT389 | | 9.31 | | MSH6 | | 13.75 | | X4E.BP1_pT37_T46 | | | 34.52 |  |  |
| STAT5.α | | 6.67 | | JNK | | 9.40 | | X53BP1 | | 16.59 | | NF2 | | | 43.09 |  |  |
| eIF4E | | 6.70 | | PTEN | | 9.48 | | X4E.BP1 | | 16.75 | | eIF4G | | | 45.24 |  |  |
| PI3K.p85 | | 6.97 | | GSK3.α.β | | 9.69 | | ACC1 | | 17.51 | | Akt. | | | 50.23 |  |  |
| PKC.α_pS657 | | 7.07 | | PRAS40_pT246 | | 9.81 | | S6_pS240_S244 | | 17.69 | | Cyclin_B1 | | | 57.31 |  |  |
| p38_MAPK | | 7.10 | | MEK1 | | 9.94 | | β.Catenin | | 19.48 | | Rb_pS807_S811 | | | 87.08 |  |  |
| mTOR_pS2448 | | 7.38 | | GSK3_pS9 | | 9.94 | | Smac | | 20.07 | | Caveolin.1 | | | 198.83 |  |  |
| MSH2 | | 7.52 | | VEGFR2 | | 10.18 | | Paxillin | | 20.16 | |  | | |  |  |  |
| HER3 | | 7.53 | | PI3K.p110.α | | 10.46 | | YB.1 | | 20.86 | |  | | |  |  |  |
| ACC_pS79 | | 7.54 | | X4E.BP1_pS65 | | 10.74 | | mTOR | | 21.76 | |  | | |  |  |  |
| eEF2K | | 8.06 | | Src_pY527 | | 10.76 | | DJ.1 | | 22.07 | |  | | |  |  |  |
| Chk2 | | 8.13 | | MAPK_pT202_Y204 | | 11.48 | | eEF2 | | 23.44 | |  | | |  |  |  |
| E.Cadherin | | 8.13 | | NF.kB.p65_pS536 | | 12.74 | | Gab2 | | 25.11 | |  | | |  |  |  |
| EGFR | | 8.22 | | p70S6K | | 12.89 | | YAP_pS127 | | 25.13 | |  | | |  |  |  |
| HER2 | | 8.54 | |  | |  | |  | |  | |  | | |  |  |  |
