# Supplementary material for: MicroRNA and Protein Profiling of Brain Metastasis Competent Cell-Derived Exosomes
Source: PLoS One. 2013 Sep 16;8(9):e73790. doi: 10.1371/journal.pone.0073790 (PMC3774795; doi:10.1371/journal.pone.0073790)
Supplement: Protocol S1 — RPPA methodology. Methodology employed by the RPPA Core Facility at MD Anderson Cancer Center (Houston, TX) to perform the Reverse Phase Protein Array. (DOCX) [file pone.0073790.s006.docx]

Proteins were denatured by 1% SDS (with β-mercaptoethanol) and diluted in five 2-fold serial dilutions in dilution buffer (lysis buffer containing 1% SDS). Serial diluted lysates were arrayed on nitrocellulose-coated slides (Grace Biolab, Bend, OR) by Aushon 2470 Arrayer (Aushon BioSystems, Billerica, MA). Total 5808 array spots were arranged on each slide including the spots corresponding to positive and negative controls prepared from mixed cell lysates or dilution buffer, respectively. Each slide was probed with a validated primary antibody plus a biotin-conjugated secondary antibody. Only antibodies with a Pearson correlation coefficient between RPPA and western blotting of greater than 0.7 were used in reverse phase protein array study. Antibodies with a single or dominant band on western blotting were further assessed by direct comparison to RPPA using cell lines with differential protein expression or modulated with ligands/inhibitors or siRNA for phospho- or structural proteins, respectively. The signal obtained was amplified using a Dako Cytomation–catalyzed system (Dako, Carpinteria, CA) and visualized by 3,3'-Diaminobenzidine (DAB) colorimetric reaction. The slides were scanned, analyzed, and quantified using a customized-software Microvigene (VigeneTech Inc., Carlisle, MA) to generate spot intensity. Each dilution curve was fitted with a logistic model (“Supercurve Fitting” developed by the Department of Bioinformatics and Computational Biology in MD Anderson Cancer Center, “http://bioinformatics.mdanderson.org/OOMPA”). This fits a single curve using all the samples (e.g., dilution series) on a slide with the signal intensity as the response variable and the dilution steps are independent variable. The fitted curve is plotted with the signal intensities – both observed and fitted - on the y-axis and the log2-concentration of proteins on the x-axis for diagnostic purposes. The protein concentrations of each set of slides were then normalized by median polish, which was corrected across samples by the linear expression values using the median expression levels of all antibody experiments to calculate a loading correction factor for each sample.
